# Elevated plasma homocysteine in type 2 diabetes mellitus: a risk factor for cardiovascular diseases

**Published:** 2012-06-24

**Authors:** Maria Onomhaguan Ebesunun, Esther Odunayo Obajobi

**Affiliations:** 1 Department of Chemical Pathology and Immunology Faculty of Basic Medical Sciences, Obafemi Awolowo College of Health Sciences Olabisi Onabanjo University Sagamu, Nigeria; 2 Department of Chemical Pathology Obafemi Awolowo Teaching Hospital Ile Ife, Nigeria

**Keywords:** Type 2 diabetes, glucose, folic acid, homocysteine, high density lipoprotein, cardiovascular disease, triglyceride, vitamin B12

## Abstract

**Background:**

Elevated plasma total homocysteine (tHcy) concentration has been associated with an increased risk for cardiovascular events in type 2 diabetic individuals independent of conventional risk factors. Available study in Nigerian-Africans is scare.

**Methods:**

Seventy (30 males) and (40 females) type 2 diabetes mellitus, with age mean of 54 ± 11.52 years were selected for this study and thirty apparently healthy volunteers were included as controls. The biochemical parameters and anthropometric indices were determined using standard procedures.

**Results:**

Significant increases were obtained in body weight, body mass index (p<0.001) and waist circumference (p<0.012) when compared with the corresponding control values respectively. The fasting plasma glucose (p<0.01), tHcy (p<0.02), and triglyceride (p<0.03) were significantly higher in the diabetes group when compared with the corresponding control values. The plasma folic acid and vitamin B_12_ (p<0.05) were significantly reduced compared to the control values. The tHcy (p<0.01) was significantly higher in the males when compared with the corresponding female value. Significant decrease was obtained in the plasma triglyceride (p<0.003) in the male patients when compared with the female patients.

**Conclusion:**

Our result showed increased plasma tHcy, triglyceride and waist circumference as well as decreased folic acid and vitamin B_12_ in type 2 diabetes mellitus. These alterations are risk factors for premature CVD events.

## Background

Increased plasma glucose leads to vascular dysfunction [1, 2], therefore diabetes have a two to four-fold greater risk of vascular disease occurrence as compared with non-diabetes [3]. In recent years, it has been suggested that atherosclerosis develops even in the presence of mild impairment of glucose tolerance [4]. The prevalence rate ot Type 2 diabetics in rural Africans is 1-2% [2]. Studies [5, 6] have linked elevated plasma homocysteine with endothelial dysfunction, renal faliure, CVD among others. In addition, studies have shown positive association between homocysteine and pathophysiology of diabetes mellitus (DM) [6, 7]. Classical hyperhomocysteinuria is a rare diisease caused by deficiencies of cystastathionine ß synthetase, methylene tetrahydofolate reductase,and methionin synthetase [7, 8]. Numerous studies [9, 10] have shown that deficiencies of vitamins B6, B_12_ and folic acid are associated with increased plasma homocysteine. On the other hand, available evidence [11] has shown that regulated physical exercise can lower plasma homocysteine.

The role of alcohol consumption in raise plasma homocysteine is inconsistent, recent finding on subjects with established CVD revealed that alcohol consumption has no significant effect on plasma homocysteine [12]. There are indications that elevated plasma homocysteine can predict early cardiovascular events [12]. However, few prospective studies [13, 14] in diabetes have shown an independent association between elevated plasma homocysteine level and all-cause mortality.

An earlier study in Nigerian-Africans on established cardiovascular disease (CVD) showed a moderate increased in plasma homocysteine [12]. There is paucity of information on the relationship between plasma homocysteine, vitamin B_12_, folic acid and type 2 DM in Nigerians living along the West Coast of Africa.

This study was designed to assess the role of plasma homocysteine, vitamin B_12_ and folic acid in predicting early cardiovascular events in type 2 DM in Nigerian-Africans. In this study plasma homocysteine, vitamin B_12_ folic acid, lipids and lipoproteins were examined in type 2 diabetes mellitus patients.

## Methods

Seventy patients (30 males) and (40 females) type 2 diabetes (1.5:2) and age range of 45-75 years with mean age of 54±11.52 years, attending the Medical Outpateint Clinic were randomly selected. The diagnosis of type 2 DM was assessed by the attending Consultant Physican using clinical history and fasting plasma glucose (FPG) =7.0mmol/L (=126mg/dl) and casual plasma glucose (random plasma glucose) of >11.0mmol/L (=200mg/dl) or the 2-hour plasma glucose of >11.0mmol/L after a 75g oral glucose load. A standard structured questionnaire was administered to each patient. A written or oral informed consent was obtained from each patient to participate in the study. Inclusion criteria were strictly obese type2 DM with age >45 years.

Type 1 diabetes mellitus or patients with liver, renal diseases and any other diseases that could affect the outcome of this study were excluded.

Thirty (30) apparently healthy free living ideal body weight volunteers consisting of twelve (12) males and eighteen (18) females (1:1.5) age range of 45-65years with mean age of 49±11.7 years were randomly selected and included as controls. All volunteers had detailed medical examinations/tests to exclude diabetes, liver disease, renal dysfunction and any other ailments that may affect the outcome of this study. Questionnaire was also administered to each volunteer.

Ethical approval was obtained from Medical Ethics and Research Committee of the Obafemi Awolowo University Teaching Hospital Ile Ife.

### Anthropometric measurements

The body weight of each subject was measured on Seca weighing scale. Height was taken with each subject standing against a calibrated scale. The waist circumference was measured with non stretchable tape rule and body mass index (BMI) was calculated H2/W (kg/m^2^) [15].

### Blood sample collection

All blood specimens were drawn in the morning after an overnight fast of 10- 14 hours into EDTA and fluoride oxalate bottles and these were immediately placed in ice pack bag. The blood samples were centrifuged using Livingstone centrifuge model LS 90-2 (manufactured by Livingstone Medicals England), the plasma samples were stored at -20°C until analyzed for tHcy, lipids and lipoproteins while the fluoride oxalate samples were analyzed for glucose within few hours of collection.

### Biochemical analysis

Plasma total cholesterol and triglyceride levels were determined enzymatically using the methods of Allain et al, [16] and Fossati [17] respectively. Plasma high-density lipoprotein cholesterol level was determined enzymatically after precipitating out low-density lipoproteins and very-low-density lipoproteins with dextran MgCl and the resulting supernatant was determined for HDLC [16]. Plasma low density lipoprotein was calculated using Freidwald et al, formula [18]. LDLC=TC-(TG/5+HDLC).

Plasma total homocysteine was determined using Axis Homocysteine Enzyme Immunoassay kit method [19]. Plasma vitamin B_12_ and folic acid were determined. using HPLC. Plasma glucose was determined using glucose oxidase method [20].

Accuracy and precision of biochemical tests were monitored by including commercial quality control samples within each batch of test assay.

### Statistical Analyses

All results were subjected to statistical analyses using SPSS for Windows, version 10.0. The results were expressed mean ± SD. Differences between means were assessed using the studentt t-test for independent samples. Post Hoc test was also performed. Two –tailed independent t- test of significance at 95% confidence limit p value less than 0.05(p

## Results

Table 1 shows anthropometric indices in patients and controls. There were significant increases in body weight and BMI (p<0.001) as well as waist circumference (WC) (p<0.012) when compared with the corresponding control values respectively.

Table 2 shows the mean ± standard deviation of plasma glucose, total homocysteine, lipids, lipoproteins, as well as folic acid and vitamin B_12_ in patients and controls. The fasting plasma glucose (p<0.01), tHcy (p<0.02), and TG (p<0.03) were significantly higher, while plasma folic acid and vitamin B_12_ (p<0.001) were significantly decreased in the DM group when compared with the corresponding control values. There were no significant changes in the other parameters.

**Figure 1:**
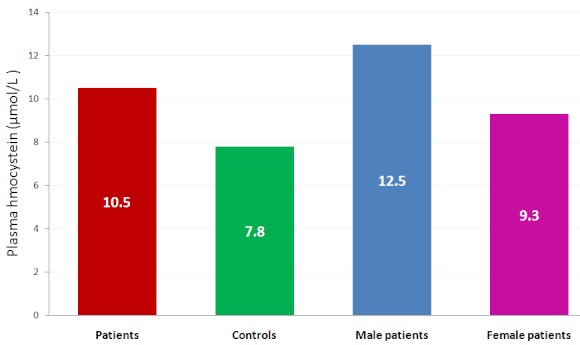
Plasma homocysteine levels in patients, controls, male and female patients

Table 3 shows the mean ± SD of plasma glucose, total homocysteine, lipids, lipoproteins, folic acid and vitamin B_12_ in male and female patients. The plasma tHcy (p<0.01) and folic acid (p<0.05) were significantly higher in the male when compared with the corresponding female values. Significant decreases were obtained in plasma TG (p<0.003) and vitamin B_12_ (p<0.05) in the male patients when compared with the female patients. No significant changes were obtained in the other parameters. Figure 1 shows the mean plasma tHcy levels of the diabetes mellitus and control subjects based on gender. The type 2 diabetes have a higher mean value of 10.5µmol/L compared with the corresponding controls of 7.7 µmol/L. The male patients have a higher mean value of 12.2 µmol/L while the female patients have 9.6 µmol/L.

**Table 1:** Anthropometric indices in patients and controls (mean + S.D)

Variables	Patients	Controls	t-test	p-value
	n= 70	n= 30		
Body weight (kg)	72+ 11.8	62+ 9.5	3.688	p < 0.001
Height (m)	1.54+ 0.98	1.6+ 0.11	2.342	p < 0.03
BMI (kg/m^2^)	30+ 4.8	24+ 3.7	5.584	p < 0.001
WC (cm)	93+ 16.2	84+ 8.8	2.594	p < 0.012

Kg= kilogram; m= metre; BMI= Body Mass Index; Kg/m^2^=kilogram/ metre^2^; WC= waist circumference; Cm= centimetre

**Table 2:** Plasma glucose, total homocysteine, lipids, lipoproteins folic acid and vitamin B_12_ in patients and controls

Variables	Patients	Controls	t-value	p-value
	n= 70	n= 30		
FPG (mmol/L)	7.81±4.04	4.76±0.9	3.922	0.01
tHcy (µmol/L)	10.51±3.92	7.71±2.87	3.232	0.02
TC (mmol/L)	5.46±2.14	4.91±0.71	1.318	ns
TG (mmol/L)	1.02±0.41	0.75±0.24	3.098	0.03
HDLC (mmol/L)	1.53±0.43	1.59±0.45	0.501	ns
LDLC (mmol/L)	2.87±0.96	2.88±0.71	0.037	ns
HDLC/TC	0.31±0.1	0.33±0.1	0.738	ns
LDLC/TC	0.55±0.13	0.58±0.1	1.251	ns
Folic acid (µg/L)	52.65±0.98	59.34±1.09	−3.88	p < 0.001
Vitamin B_12_ (µg/L)	48.47±0.74	58.48±1.51	−6.61	p < 0.001

FPG = Fasting Plasma Glucose; tHcy= Total Homocysteine; TC= Total Cholesterol; TG= Triglyceride; HDLC= High Density Lipoprotein Cholesterol; LDLC= Low density Lipoprotein Cholesterol; mmol/L= millimol per litre; µmol/L= micromol per litre; ns= not significant

**Table 3:** Plasma glucose, total homocysteine, lipids, lipoproteins folic acid and vitamin B_12_ in male and female diabetes mellitus patients.

Variables	Males	Females	t-value	p-value
	n= 30	n= 40		
FPG (mmol/L)	8.81±4.6	7.2±3.6	1.24	NS
tHcy (µmol/L)	12.5±4.7	9.6±2.8	2.76	0.01
TC (mmol/L)	4.8±1.0	5.9±2.6	1.53	NS
TG (mmol/L)	0.89±0.2	1.2±0.5	3.23	0.03
HDLC (mmol/L)	1.6±0.4	1.5±0.4	0.363	NS
LDLC (mmol/L)	2.8±1.0	2.9±0.9	0.541	NS
BMI (kg/m^2^)	29.0±4.1	31.0±5.0	1.378	NS
HDLC/TC	0.34±0.11	0.29±0.10	1.491	NS
LDLC/HDLC	0.56±0.12	0.54±0.14	0.461	NS
Folic acid(µg/L)	53.64±1.36	51.82±1.40	2.21	0.05
Vitamin B_12_ (µg/L)	47.72±1.16	49.09±0.95	2.50	0.05

FPG = Fasting Plasma Glucose; tHcy= Total Homocysteine; TC= Total Cholesterol; TG= Triglyceride; BMI= Body Mass Index; HDLC= High Density Lipoprotein Cholesterol; LDLC= Low density Lipoprotein Cholesterol; Ns= not significant

## Discussion

To our knowledge, this is the first comprehensive study of homocysteine, folic acid and vitamin B_12_ levels in type 2 DM in Nigeria. The study subjects were predominantly obese with mean BMI value of 30±4.8kg/m^2^ when the WHO classification of BMI was applied [21]. Obesity, a predisposing factor for the development of type 2 diabetes mellitus has been linked with other health related complications [22].

Moderately raised plasma homocysteine was obtained with associated decreased folic acid and vitamin B_12_ levels in type 2 diabetes subjects. In an earlier study on type 2 diabetes, a strong association between elevated plasma homocysteine level and early CVD events was reported [23]. Increased tHcy and reduced levels of these vitamins that are essential in homocysteine metabolism as obtained in this study are indications that type 2 DM are at risk of early CVD events. The mechanisms by which homocysteine promotes cardiovascular disease are uncertain. Several studies suggest that elevated plasma homocysteine level has both atherogenic and thrombogenic effects [7, 23], causes endothelial dysfunction by increasing oxidative stress [7] and in part decreases the release of nitric oxide as well as impairing vasodilatation [23]. The data from our study are compatible with the hypothesis that a folate-sensitive defect in homocysteine metabolism contributes to depressed methylenetetrahydrofolate reductase activity, a risk factor for CVD and this risk may be ameliorated with folate and vitamin B_12_ supplementation. Several homocysteine-reducing strategies have been discovered, first for metabolic hyperhomocystinemia disorders [13] and more recently in studies for reduction of risk from cardiovascular and cerebrovascular disorder [23], folic acid, cobalamin, and pyridoxine supplementation were reported to lower plasma homocysteine. It is conceivable that daily supplementation with these vitamins could prevent clinical deterioration in some patients with CVD.

The plasma tHcy obtained in this study did not correlate with any of the measured parameters, thus supporting earlier studies [4, 24] which reported that an increase plasma tHcy level is an independent risk factor for early cardiovascular disease mortality in type 2 diabetes patients.

There was also gender variation in the mean plasma tHcy with higher value in the males than the females. This difference persisted even after adjusting for age. This cofounder may be the factor responsible for the vulnerability of the males to premature CVD risk than the females. Reports from earlier studies have also substantiated the present finding that men tend to have higher levels of plasma tHcy than women [22, 25].

Since homocysteine stimulates oxidative stress and inhibits nitric oxide formation [7, 26], increased plasma homocysteine as observed in type 2 diabetic patients in the present study could promote platelet hyperactivity. It therefore suggests that some of the indicators of CVD risk factor associated with mortality in DM patients may be elevated plasma homocysteine and reduced folic acid and vitamin B_12_.

There were some degrees of dyslipidemia in our patients compared to the control group, with significantly higher plasma TG in the diabetes. This change could be as a result of mobilization of free fatty acids to the liver which can lead to increase synthesis of TG and in turn gives rise to pancreatic lipotoxicity in type 2 diabetes mellitus patients [27]. Increased oxidative stress has also been reported [24, 25] in type 2 DM and this could cause matrix alteration, thereby making DM patients more susceptible to the effect of increased plasma triglyceride.

Although studies [11, 12] have indicated elevated plasma homocysteine as a strong CVD risk factor, no available report in Nigeria has linked increased plasma homocysteine, decreased vitamin B_12_ and folic acid with type 2 diabetes mellitus. The result from our study has showed that increased plasma homocysteine, decreased vitamin B_12_ as well as folic acid could be used as possible predicting risk factors for premature cardiovascular events in type 2 DM.

## Conclusion

The main findings from our study are increased plasma homocysteine, triglyceride and waist circumference, decreased folic acid and vitamin B_12_ in type 2 diabetes mellitus. The changes are indications of possible increase risk of premature cardiovascular disease. Supplementation with folic acid and vitamin B_12_ in addition to regular treatment will be beneficial to type 2 diabetes patients to prevent possible early CVD events. However, further study will be warranted to ascertain the present findings.
